# The use of cellular thermal shift assay (CETSA) to study Crizotinib resistance in ALK-expressing human cancers

**DOI:** 10.1038/srep33710

**Published:** 2016-09-19

**Authors:** Abdulraheem Alshareef, Hai-Feng Zhang, Yung-Hsing Huang, Chengsheng Wu, Jing Dong Zhang, Peng Wang, Ahmed El-Sehemy, Mohamed Fares, Raymond Lai

**Affiliations:** 1 Department of Laboratory Medicine and Pathology, University of Alberta, Edmonton, Alberta, Canada; 2 Department of Applied Medical Sciences, Taibah University, Almedinah, P.O. Box 41477, Saudi Arabia; 3 Department of Pathology and Laboratory Medicine, University of British Columbia, Vancouver, BC V5Z 1L3, Canada; 4 Department of Medical Oncology, The First hospital of China Medical University, Shen Yang 110001, P. R. China; 5 Department of Internal Medicine, University of Alberta, Edmonton, Alberta, Canada; 6 Department of Laboratory Medicine and Pathobiology, University of Toronto, Toronto, Canada; 7 National Research Center, Cairo, Egypt; 8 Department of Oncology, University of Alberta, Edmonton, Alberta, Canada; 9 DynaLIFEDx Medical Laboratories, Edmonton, Canada

## Abstract

Various forms of oncogenic ALK proteins have been identified in various types of human cancers. While Crizotinib, an ALK inhibitor, has been found to be therapeutically useful against a subset of ALK^+^ tumours, clinical resistance to this drug has been well recognized and the mechanism of this phenomenon is incompletely understood. Using the cellular thermal shift assay (CETSA), we measured the Crizotinib—ALK binding in a panel of ALK^+^ cell lines, and correlated the findings with the ALK structure and its interactions with specific binding proteins. The Crizotinib IC_50_ significantly correlated with Crizotinib—ALK binding. The suboptimal Crizotinib—ALK binding in Crizotinib-resistant cells is not due to the cell-specific environment, since transfection of *NPM-ALK* into these cells revealed substantial Crizotinib—NPM-ALK binding. Interestingly, we found that the resistant cells expressed higher protein level of β-catenin and siRNA knockdown restored Crizotinib—ALK binding (correlated with a significant lowering of IC_50_). Computational analysis of the crystal structures suggests that β-catenin exerts steric hindrance to the Crizotinib—ALK binding. In conclusion, the Crizotinib—ALK binding measurable by CETSA is useful in predicting Crizotinib sensitivity, and Crizotinib—ALK binding is in turn dictated by the structure of ALK and some of its binding partners.

*Anaplastic lymphoma kinase (ALK*), which encodes a tyrosine kinase member of the insulin receptor superfamily, was initially discovered and characterized as one of the two fusion gene partners identified in anaplastic large-cell lymphoma (ALCL) carrying the *t*(*2; 5*) chromosomal abnormality^1^. In ALCL, the catalytic domain of the ALK protein was fused with the amino terminus of nucleophosmin (NPM), and it was found that the NPM-ALK fusion protein results in constitutive activation of the ALK tyrosine kinase, thereby leading to deregulation of multiple cell signalling pathways and increased tumorigenicity^2^. Subsequent studies of ALCL and other types of human cancer have revealed various types of *ALK* gene aberrations and additional fusion partners of ALK^3^,^4^. For instance, the echinoderm microtubule-associated protein like 4 (*EML4*)-*ALK* fusion was identified in a small subset of non-small cell lung cancers (NSCLC)^5^,^6^. In neuroblastoma (NB), the most common and aggressive childhood malignancy, *ALK* has been found to be amplified or mutated at various locations^7^,^8^,^9^,^10^,^11^. The presence of any *ALK* aberrations in NB correlates with a short overall survival^12^. In keeping with the pathogenetic importance of ALK, inhibition of ALK using pharmacologic agents or siRNA has been shown to result in cell cycle arrest and apoptosis in various forms of ALK-positive (ALK^+^) human cancers^13^.

Crizotinib is the first ALK inhibitor used in the clinic and it has demonstrated remarkable efficacy against ALK^+^ tumours occurring in mouse models as well as humans^3^. For instance, Crizotinib has shown remarkable anti-tumour activity in relapsed ALK^+^ALCL patients^14^,^15^. However, while Crizotinib has been shown to be therapeutically efficacious in treating ALK^+^ NSCLC patients, many of the treated patients showed disease progression within a year of therapy^16^. A number of recent studies have demonstrated that the therapeutic benefits of Crizotinib are variable among different types of ALK^+^ cancer^12^,^17^. The mechanisms underlying the differential clinical responses to Crizotinib are not well understood. Initial studies in small cohorts of patients have already shown that mutations within the *ALK* kinase domain can drive acquired resistance to Crizotinib^3^,^18^. In NSCLC, while it was initially reported that the differential Crizotinib sensitivity in EML4-ALK-expressing cells is related to the existence of the four EML4-ALK fusion variants^19^, results from subsequent studies did not confirm the relationship between these EML4-ALK variants and Crizotinib responses^17^,^20^. In a phase 1 clinical trial, a wide range of Crizotinib responsiveness was found in a cohort of ALK^+^ neuroblastoma patients^14^. Taken together, resistance to Crizotinib remains to a significant challenge in the clinic, and the mechanisms underlying this specific drug resistance is incompletely understood.

In this study, we aimed to study the biology of Crizotinib resistance, by correlating various forms of ALK in a panel of ALK^+^ cancer cell lines and the *in vitro* sensitivity to Crizotinib. We hypothesize that the physical binding between Crizotinib and ALK is the determining factor of Crizotinib sensitivity, and thus, the extent of Crizotinib—ALK binding can be used to predict the biological response to Crizotinib. To quantitatively measure the Crizotinib—ALK binding, we employed the cellular thermal shift assay (CETSA), a recently described method that allows rapid and simple assessment of target engagement of drugs in a cellular context^21^,^22^,^23^. Our results have led us to conclude that the Crizotinib—ALK binding measurable by CETSA is useful in predicting Crizotinib sensitivity in ALK^+^ cancer cells, and Crizotinib—ALK binding is in turn dictated by structure of ALK and some of its binding partners.

## Results

### Crizotinib—ALK binding correlates with Crizotinib sensitivity in ALK-expressing cells

First, we asked if there is a correlation between Crizotinib—ALK binding and Crizotinib sensitivity in ALK-expressing cells. To answer this question, we performed CETSA using 7 ALK-expressing cell lines, including 2 ALK-positive anaplastic large cell lymphoma (ALK^+^ALCL) cell lines (Karpas 299 and SupM2), 4 neuroblastoma cell lines (NB1, IMR32, GOTO and SK-N-SH) and one non-small cell lung cancer cell line (H2228), and correlated these results with the Crizotinib sensitivity (i.e. inhibitory concentration at 50%, IC_50_). The expression of the ALK proteins and their phosphorylation status in these 7 cell lines are illustrated in Supplementary Figure 1. In the left panel in which the results from the 4 neuroblastoma cells lines are illustrated, we found the 220 kDa band, which represents the full-length ALK protein, and/or several bands at lower molecular weight (e.g. 140 kDa). These findings are in accordance with the published findings from other groups^24^. Western blots using anti-pALK showed essentially a similar pattern as that of anti-ALK. In the right panel where the results of the three cell lines carrying ALK fusion proteins are shown, we found the ALK fusion proteins at their expected molecular weights. Specifically, NPM-ALK present in SupM2 and Karpas 299 was located at approximately 80 kDa, whereas EML4-ALK present in H2228 was found at approximately 89 kDa, as reported previously^25^. SP53, a mantle cell lymphoma cell line, served as the negative control for ALK and pALK.

**Figure 1:**
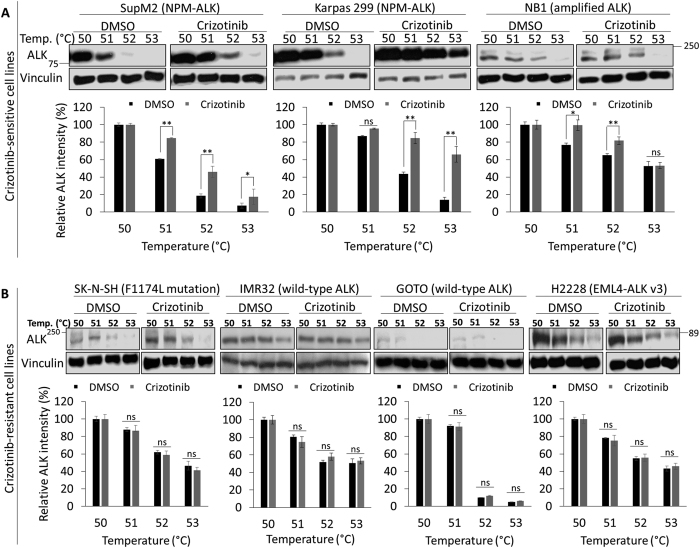
Crizotinib, an ALK inhibitor, binds to ALK in Crizotinib-sensitive cell lines but not in Crizotinib-resistant cell lines. CETSA was performed to measure the binding ability of Crizotinib to different ALK forms in Crizotinib-sensitive and Crizotinib-resistant cell lines. (**A)** Three Crizotinib-sensitive cell lines (i.e. SupM2, Karpas 299 and NB1) were treated with 50 nM Crizotinib for 6 hours. Representative ALK Western blots of each cell line are shown on the upper panel. (**B**) Four Crizotinib-resistant cells (i.e. SK-N-SH, IMR32, GOTO and H2228) were treated with 2000 nM Crizotinib for 6 hours. Representative ALK Western blots of each cell line are shown on the upper panel. Full-length blots are presented in Supplementary Figure 11. Vinculin level was blotted as a loading control. Data are presented as mean ± SD. *P < 0.05, **P < 0.01, Student’s *t* test.

CETSA results and the IC_50_ data (derived from the literature as well as our own studies) are summarized in Table 1^26–30^. For the purpose of this study, cell lines with an IC_50_ of ≤56 nM, including the two ALK^+^ALCL cell lines and NB1, were considered Crizotinib-sensitive; the other 4 cell lines that carried an IC_50_ of >56 nM were considered Crizotinib-resistant. Results from CETSA are illustrated in Fig. 1A,B. As detailed in Materials and Methods, Crizotinib—ALK binding was assessed ‘positive’ if there was significantly more ALK in the Crizotinib-treated group compared to the DMSO-treated group at 52 °C detectable by Western blots. In comparison, Crizotinib—ALK binding was assessed ‘negative’ if there was no significant difference in the ALK expression level between the two groups at 52 °C. Statistical analysis using Fisher exact test has revealed that the correlation between Crizotinib sensitivity and Crizotinib—ALK binding among these 7 cell lines is significant (*P* = 0.029). Overall, these results suggest that a lack of Crizotinib—ALK binding is a major contributing factor to Crizotinib resistance in ALK-expressing cancer cells.

### Differential Crizotinib—ALK binding is dictated by the ALK structure

We then asked if increasing the concentration of Crizotinib will promote Crizotinib—ALK binding in Crizotinib-resistant cell lines. As shown in Supplementary Figure 2, increasing the concentrations of Crizotinib in two Crizotinib-sensitive cell lines (SupM2 and NB1) resulted in an appreciable increase in the stabilization of ALK at 52 °C. In contrast, increasing the concentrations of Crizotinib in three Crizotinib-resistant cell lines (SK-N-SH, IMR32 and H2228) consistently failed to yield any detectable change to the ALK stabilization at 52 °C. Correlating with these findings, we noted that the viability of both sensitive cell lines dropped by an average of 65% when the Crizotinib concentrations at their IC_50_’s were doubled. In contrast, the viability of the three resistant cell lines dropped by an average of only 30% when the Crizotinib concentrations at their IC_50_’s were doubled (not shown). Taken together, these findings further support the concept that a lack of Crizotinib—ALK binding is a major contributing factor to Crizotinib resistance in ALK-expressing cancer cells.

**Figure 2:**
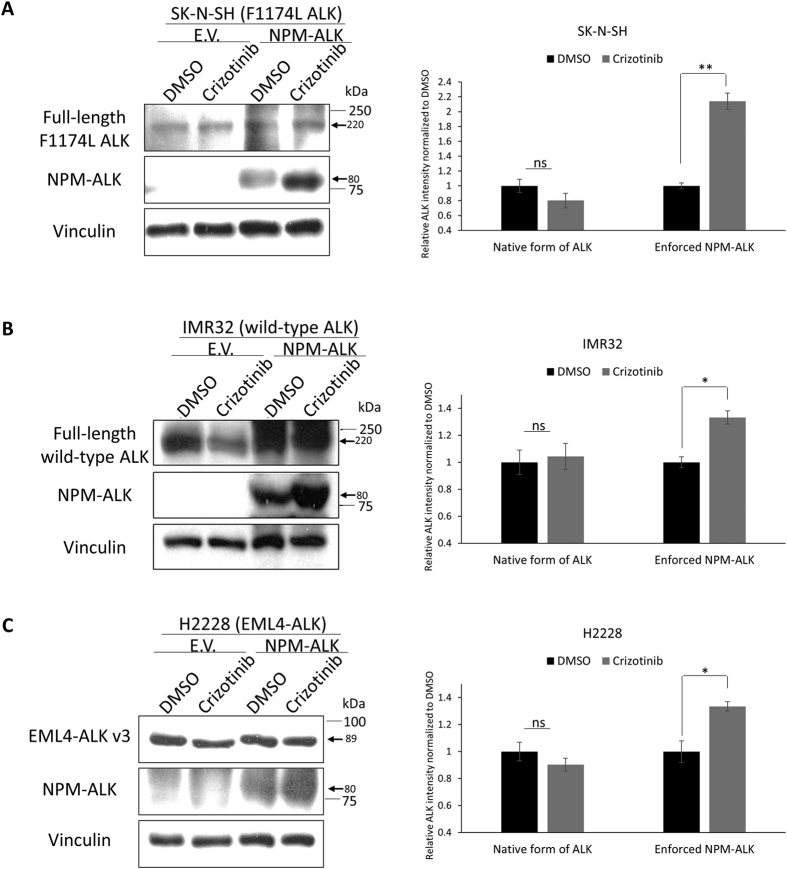
Differential sensitivity to Crizotinib, through physical binding, is specific to ALK structure. **(A–C)** Enforced expression of *NPM-ALK* into Crizotinib-resistant cell lines (i.e. SK-N-SH, IMR32, and H2228) were treated with 50 nM Crizotinib for 6 hours and showed stabilization of NPM-ALK but not the native ALK. CETSA assay was performed at 52 °C. Representative Western blots are shown on the left side and the densitometry quantification data from 3 independent experiments are shown on the right side. Data are presented as mean ± SD. *P < 0.05, **P < 0.01, Student’s *t* test.

To assess whether the differential Crizotinib—ALK binding among different cell lines is due to the cell-type specific biochemical background and/or a difference in the efficiency of Crizotinib transport into the cells, we transiently transfected *NPM-ALK* into 3 Crizotinib-resistant cell lines (SK-N-SH, IMR32 and H2228). By CETSA, we found substantial Crizotinib-NPM-ALK binding (Fig. 2). In the same experiments, there was no substantial binding between Crizotinib and the native forms of ALK. These results support the concept that the differential Crizotinib sensitivity and Crizotinib—ALK binding is greatly determined by the structure/biochemistry of ALK structure, but not related to a lack of Crizotinib transport into the cells or specific cell-type specific biochemical environment.

To further substantiate these findings, we performed additional experiments in which 3 different forms of ALK (NPM-ALK, full-length wild-type ALK and full-length, mutated ALK^F1174L^) were transfected into GP293 cells (Supplementary Figure 3). As shown in Supplementary Figure 4, only NPM-ALK was bound by Crizotinib and its pALK signals were largely abrogated with Crizotinib treatment. In contrast, the same treatment in cells transfected with the full-length wild-type *ALK* or *ALK*^*F1174L*^ did not result in any substantial change to the ALK stabilization and only a partial decrease in pALK. Thus, these results also support the concept that the ALK structure is an important determinant of the Crizotinib—ALK binding.

### Abrogation of Crizotinib—ALK binding in Crizotinib-resistant ALK^+^ALCL cell lines

To reinforce the concept that the Crizotinib—ALK binding pattern revealed by CETSA is useful in predicting Crizotinib sensitivity, we established two Crizotinib-resistant ALK^+^ALCL cell clones derived from Karpas 299 and SupM2. These cell clones were established by subjected them to increasing concentrations of Crizotinib over a few weeks, reaching a final concentration of 500 nM for both cell lines.

**Figure 3:**
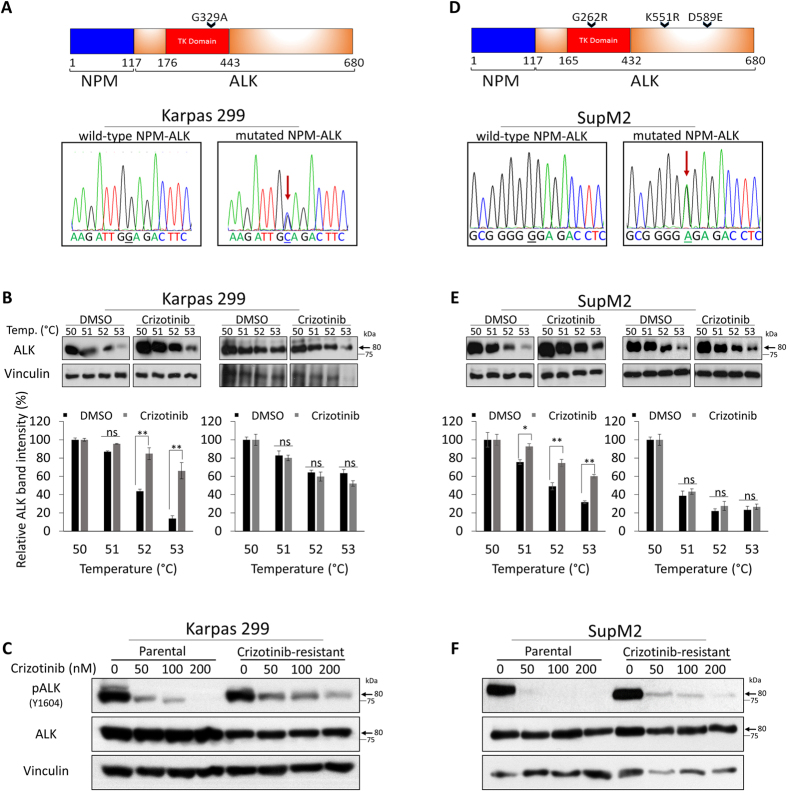
Identification of NPM-ALK secondary mutations in Crizotinib-resistant ALK^+^ALCL cell lines that abrogate the binding between Crizotinib and ALK. (**A**) Sequencing of ALK kinase domain coding fragment in Crizotinib-sensitive and Crizotinib-resistant ALK^+^ALCL cell lines. Schematic of ALK kinase domain mutations associated with acquired resistance to Crizotinib. (**A,D**) Show the electropherograms of NPM-ALK cDNA from parental Karpas 299, Crizotinib-resistant Karpas 299, parental SupM2, and Crizotinib-resistant SupM2 cells. (**B,E**) Show that Crizotinib—ALK binding can be detected using CETSA assay in Crizotinib-sensitive cells but not in Crizotinib-resistant clones (full-length blots are presented in Supplementary Figure 12). (**C,F**) Show that Crizotinib treatments could substantially inhibit pALK in Crizotinib-sensitive cells, while only partially inhibit pALK in Crizotinib-resistant clones.

To understand the mechanism of resistance in these Crizotinib-resistant cell clones, we sequenced the *NPM-ALK* mRNA expressed in these cells; specifically, the segment between exon 20 to exon 29 from the *ALK* domain, which includes the kinase domain, was examined. As shown in Fig. 3, four secondary mutations were identified in Crizotinib-resistant ALK^+^ALCL cell lines, with one mutation (G329A) occurring in Karpas 299 cells while 3 mutations (G262R, K551R and D589E) were observed in SupM2. The mutations at G329 (equivalent to G1269 in the full-length ALK) and G262 (equivalent to G1202 of the full-length ALK) are located in the tyrosine kinase domain. In accordance with our hypothesis, Crizotinib—ALK binding was detectable only in Crizotinib-sensitive, parental cell clones but not Crizotinib-resistant cell clones. All of these acquired Crizotinib-resistant cell lines showed a substantially lesser degree of pALK inhibition upon Crizotinib treatment (Fig. 3). To our knowledge, this is the first evidence supporting the concept that secondary mutations of ALK contribute to Crizotinib resistance by abrogating Crizotinib—ALK binding. These results further support that the ALK structure (e.g. mutations) is an important determinant of the Crizotinib—ALK binding and Crizotinib sensitivity.

To provide further support that the observed secondary mutations of ALK seen in the Crizotinib-resistant cell clones are relevant to their Crizotinib resistance, we performed two experiments. First, we found that these Crizotinib-resistant cell clones developed resistance only to Crizotinib but not Ceritinib (another ALK inhibitor) or doxorubicin (Supplementary Figure 5). These findings argue against the existence or significance of other non-ALK factors. Second, as shown in Supplementary Figure 5E, siRNA knockdown of ALK resulted in a similar and dramatic reduction in cell viability in both the parental cells and Crizotinib-resistant cell clones. These results support the central role of ALK, but not other oncoproteins, in the Crizotinib-resistant cell clones.

### The role of β-catenin in modulating Crizotinib—ALK binding and Crizotinib-resistance

**Figure 4:**
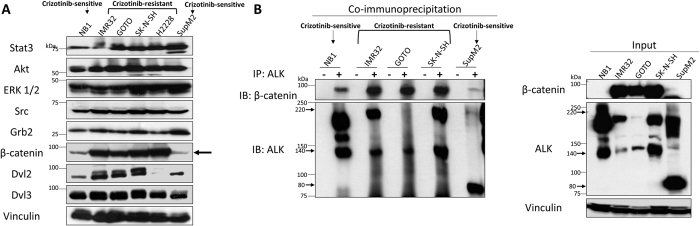
β-catenin interacts with ALK and shows of higher expression levels in Crizotinib-resistant cell lines. **(A)** Screening of a panel of known ALK-effector proteins identified β-catenin as a protein that was highly expressed in Crizotinib-resistant cell lines (i.e. IMR32, GOTO, SK-N-SH, and H2228). Please note that the Western blot from Supplementary Figure 1 was analysed here against indicated antibodies. (**B**) Left panel, ALK pull-down experiment showed substantial ALK-β-catenin binding only in Crizotinib-resistant cell lines (i.e. IMR32, GOTO and SK-N-SH). Right panel, the input for the co-immunoprecipitation.

It has been published that ALK has a large number of binding proteins^31^. We hypothesized that some of these binding proteins might play a role in modulating Crizotinib—ALK binding and Crizotinib resistance. To this end, we compared the expression levels of various known ALK-binding proteins between Crizotinib-resistant and Crizotinib-sensitive cell lines. As shown in Fig. 4A, we found that β-catenin was expressed higher in all 4 resistant cell lines (IMR32, GOTO, SK-N-SH and H2228) as compared to two sensitive cell lines (SupM2 and NB1). Two of our previous studies have shown that Karpas 299 cells expressed a similar level of β-catenin as SupM2 32,33. As shown in Fig. 4B, by immunoprecipitation, the ALK—β-catenin interaction was detectable in all cell lines examined, although the level of β-catenin pulled down with ALK was substantially higher in the three Crizotinib—resistant cell lines when compared to the Crizotinib-sensitive cell lines. This difference is highlighted when we compared GOTO (which expressed a relatively low level of ALK but a high level of β-catenin pull-down) with NB1 or SupM2 (relatively high level of ALK but a low level of β-catenin pull-down).

**Figure 5:**
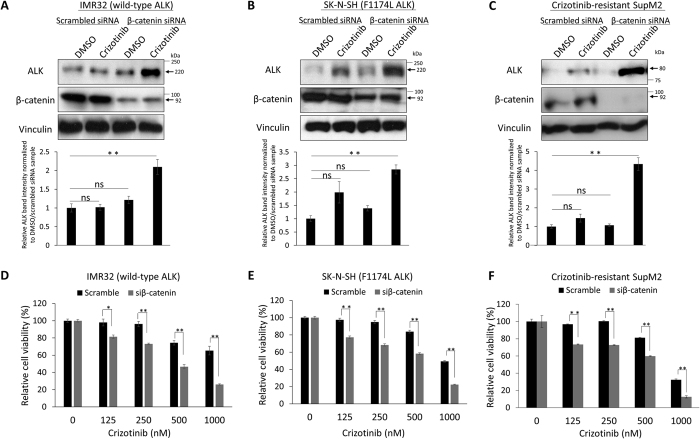
β-catenin siRNA knockdown restores Crizotinib—ALK binding and significantly sensitizes Crizotinib-resistant cell lines to Crizotinib treatment. (**A–C**) show that β-catenin siRNA knockdown significantly restored Crizotinib—ALK binding upon Crizotinib treatment in comparison to scrambled siRNA treatment. (**D–F**) show that β-catenin siRNA knockdown significantly sensitized Crizotinib-resistant cells to Crizotinib treatment in comparison to scrambled siRNA treated cells. Data are presented as mean ± SD. *P < 0.05, **P < 0.01, Student’s *t* test.

In keeping with the concept that β-catenin is important in regulating Crizotinib—ALK interaction and Crizotinib resistance, we subjected two Crizotinib-resistant cell lines (IMR32 and SK-N-SH) to β-catenin siRNA knockdown for 72 hours, and we performed CETSA assay. As shown in Fig. 5, Crizotinib stabilized ALK upon β-catenin siRNA knockdown as compared to the negative controls. Importantly, restoration of Crizotinib—ALK binding induced by β-catenin knockdown significantly sensitized IMR32 and SK-N-SH to Crizotinib, lowering the IC_50_ from 1220 nM (scrambled siRNA) to 467 nM (i.e. a 62% decrease) and from 764 nM to 336 nM (i.e. a 57% decrease), respectively. Of note, β-catenin siRNA knockdown alone (for 72 hours) did not significantly affect the cell growth of both cell lines (Supplementary Figure 6). To substantiate these finding, we repeated the same experiment using our generated Crizotinib-resistant SupM2 cell clone. As shown in Fig. 5C, these cells were sensitized to Crizotinib upon β-catenin siRNA knockdown, with the IC_50_ lowered from 1182 nM (scrambled siRNA) to 456 nM (i.e. a 62% decrease). Lastly, we transfected *EML4-ALK* and *ALK*^*F1174L*^ into NB1, a Crizotinib-sensitive cell line that expressed a relatively low level of β-catenin. As shown in Supplementary Figure 7, these two ALK forms were found to be stabilized in NB1 cells at 52 °C by CETSA. This finding is in contrast with the observation that these two ALK forms were not stabilized at 52 °C in their respective native cell lines (Fig. 1B) (i.e. H2228 and SK-N-SH, respectively).

To prove the specificity of β-catenin, we examined another known ALK-binding protein, namely HSP90, which is a chaperone reported to play an important role in protein folding^34^. The choice of using HSP90 is also related to the fact that HSP90 inhibitor has been shown to be highly effective against ALK^+^ lung cancer cells as well as ALK^+^ALCL cells in preclinical and clinical studies^35^,^36^. As shown in Supplementary Figure 8, treatment of two Crizotinib-resistant cell lines (SK-N-SH and H2228) with increasing doses of an HSP90 inhibitor (NVP-AUY922) did not substantially alter the Crizotinib—ALK binding or the Crizotinib susceptibility in the Crizotinib-resistant cell lines, lowering the IC_50_ from 752 nM (scrambled siRNA) to 60 nM (i.e. an 11% decrease) in SK-N-SH and from 974 nM to 836 nM (i.e. a 15% decrease) in H2228.

### β-catenin expression level positively correlates with the Crizotinib responsiveness

To further support the concept that β-catenin can influence the ability of Crizotinib to bind to ALK, we sought to find out if there is a correlation between the β-catenin expression level and Crizotinib responsiveness among various ALK-expressing cell lines published in the *Cancer Cell Line Encyclopedia* (CCLE) project database^30^. As illustrated in Supplementary Figure 9, it is evident that a high β-catenin mRNA level significantly correlates with the Crizotinib sensitivity (i.e. IC_50_) based on this analysis (Spearman’s correlation, R = 0.7, p = 0.029).

### β-catenin physically interferes with Crizotinib—ALK binding

**Figure 6:**
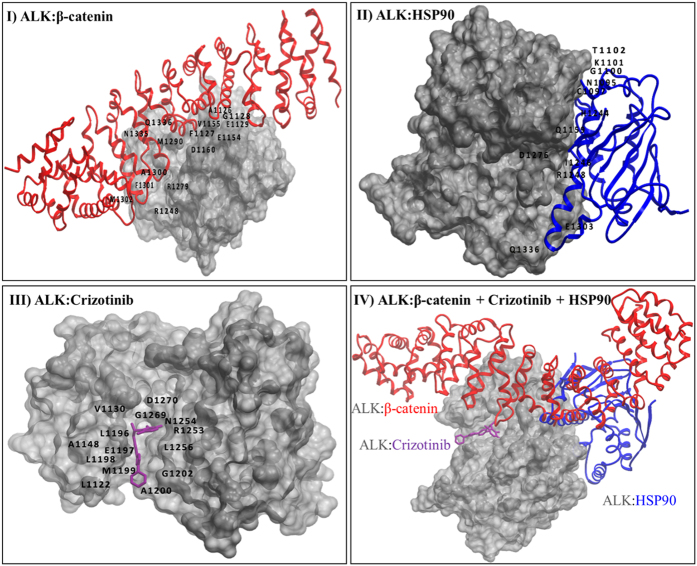
Computational analysis of ALK-β-catenin interaction supports the observed blockage of Crizotinib—ALK binding. (**I)** β-catenin was predicted to interact with 15 ALK residues (A1126, F1127, G1128, E1129, E1154, V1155, D1160, R1248, R1279, M1290, A1300, F1301, M1302, N1335 and Q1336). (**II)** HSP90 was predicted to interact with 12 ALK residues (N1095, C1097, G1100, K1101, T1102, Q1159, H1244, I1246, R1248, D1276, E1303, and Q1336). (**III)** Crizotinib was reported to bind to 14 ALK residues (L1122, V1130, A1148, L1196, E1197, L1198, M1199, A1200, G1202, R1253, N1254, L1256, G1269, and D1270). (**IV)** Interaction of ALK with β-catenin, Crizotinib and HSP90.

In view of our findings that a knockdown of β-catenin can restore Crizotinib—ALK binding and the β-catenin expression level significantly correlates with the Crizotinib IC_50_, we hypothesized that the binding between β-catenin and ALK blocks that of Crizotinib and ALK. In other words, we predicted that there is a substantial overlap in the ALK binding sites for β-catenin and ALK. Thus, based on the X-ray crystal structure of ALK published by Cui *et al*.^37^, we modeled the Crizotinib—ALK binding as well as the β-catenin—ALK binding using ClusPro docking software (Boston University), analyzed and visualized the resulting models using the Molsoft, PyMol as well as Moe software programs (as detailed in the Supplementary Information). As shown in Fig. 6, β-catenin was predicted to interact with 15 ALK residues, 7 of which (A1126 to E1129, E1154, V1155 and D1160) reside in close proximity to or surrounding the two ALK residues known to be crucial to the binding of Crizotinib, namely a G-rich loop residue (L1122) and a conserved hydrophobic residue (V1130). Additional three residues out of the 15 residues (R1248, R1279 and M1290) were also localized near to another Crizotinib-binding residue, D1270. These results suggest that β-catenin binding to ALK will exert substantial impact on Crizotinib—ALK binding. Specifically, the presence of β-catenin will likely prevent the Crizotinib molecules from reaching the targeted ALK residues or disrupt its binding with certain ALK residues. In keeping with the observation that inhibition of HSP90 did not significantly alter the IC_50_ of Crizotinib, and as a validation of the curability of our modeling and docking procedure, our prediction showed that all 12 ALK residues implicated in binding to HSP90 are located relatively remote from the ALK residues for binding Crizotinib.

## Discussion

The advent and application of specific ALK inhibitors have significantly improved the clinical outcome of patients with ALK^+^ tumours, which include (most notably) ALK^+^ALCL and ALK^+^ lung cancers^14^. Crizotinib is the first in the class of ALK inhibitors. In two clinical studies, Crizotinib used as a single agent has shown remarkable anti-tumour activity in relapsed ALK^+^ALCL patients^14^,^15^. Unfortunately, based on the results of a number of other clinical studies, resistance to ALK inhibitors occurs relatively frequently^3^,^38^,^39^. While the mechanisms underlying Crizotinib resistance is incompletely understood, the acquisition of Crizotinib-induced secondary mutations is believed to be an important factor^3^. In addition to *ALK* mutations, other mechanism of resistance to ALK inhibitors include *ALK* gene amplification and activation of alternative survival signalling pathways such as that of Kras and EGFR^18^,^40^. Thus far, there are relatively few options available to overcome drug resistance of tyrosine kinase inhibitors. The key strategy has been the development of new generations of ALK inhibitors, with the hope that these drugs can bind to ALK via alternative sites that are not affected by the mutations^3^. However, the efficacy of these new inhibitors is not consistent nor predictable^41^,^42^,^43^.

Results from this study are in agreement with the previous observation that Crizotinib sensitivity is highly variable among ALK^+^ human cancer cells^12^,^19^,^44^. Using a cohort of 7 ALK^+^ cell lines that are highly variable in Crizotinib sensitivity, we studied the biological basis of Crizotinib resistance. An important observation from our studies is that of a significant correlation between the Crizotinib sensitivity and Crizotinib—ALK binding. Using 52 °C as the cut-off in the CETSA assay, we found that all 3 Crizotinib-sensitive cell lines demonstrated Crizotinib—ALK binding, in contrast with none of the 4 resistant cell lines, including the two cell lines that carry wild-type ALK (IMR32 and GOTO). Unlike most of the previously published studies of Crizotinib resistance, which focused on the correlation between *ALK* mutations and the *in vitro* sensitivity to Crizotinib, this current study has provided direct evidence highlighting the importance of the physical interaction between Crizotinib and ALK as the key determinant for Crizotinib sensitivity. It is perceivable that the interaction between Crizotinib and ALK may be modulated by at least 3 major factors: (1) the overall biochemical and biological status that are cell-type specific; (2) the 3-dimensional structure of ALK, which is in turn strongly influenced by the presence of *ALK* gene mutations and its abnormal fusions with other genes; (3) the interactions between ALK and its binding proteins, which are in turn affected by the relative affinities between ALK and specific binding proteins as well as the expression levels of specific ALK-binding proteins.

To assess the relevance of the overall biochemical/biological status of the cells, we asked if *NPM-ALK* (which exist in the two highly Crizotinib-sensitive cell lines, Karpas 299 and SupM2) enforced expressed in Crizotinib-resistant cell lines can bind Crizotinib strongly. If the biochemical/biological status of the cells plays a key role in determining Crizotinib—ALK binding, one will expect that this interaction between NPM-ALK and Crizotinib will be greatly diminished in the three Crizotinib-resistant cell lines. Our observation that NPM-ALK remained to effectively bind to Crizotinib at 52 °C in the new environment strongly argues against the importance of this factor. Moreover, these experiments also have excluded the possibility that the differential Crizotinib—ALK binding is due to substantial differences in the efficiency of the intracellular transport of Crizotinib and/or its bioavailability inside the cells. This conclusion is further supported by our observation that increasing the concentrations of Crizotinib in the tissue culture did not appreciably affect Crizotinib—ALK binding detectable by using CETSA.

There is substantial amount of evidence supporting the importance of the structure of ALK as a determinant of the interaction between ALK and ALK inhibitors. Specifically, *ALK* mutations are known to exist and believed to be a major mechanism of the clinical resistance of ALK inhibitors^3^. In the field of ALK^+^ALCL, we are aware of only two publications describing ALK mutations in cell lines expressing NPM-ALK, and these mutations do not overlap with the mutations identified in this study^45^,^46^. In the current study, we also found two mutations at the tyrosine kinase domain of ALK, namely G329A (or G1269A) and G262R (or G1202R) in the resistant clones of Karpas 299 and SupM2, respectively. The clinical significance of these two mutations is substantiated by the observation that they have been found in tumours samples from ALK^+^ lung cancer patients^3^.

A good number of studies have been previously published in explaining how ALK mutations result in resistance to ALK inhibitors such as Crizotinib. As mentioned above, one of the mechanisms is related to the relatively high efficiency of ATP recruitment by some ALK mutants, thereby minimizing the inhibitory effect of Crizotinib^18^,^44^. In support of this concept, a study using different EML4-ALK constructs mutated at various sites of the ALK tyrosine kinase domain has concluded that these ALK mutations frequently result in increased ATP-ALK binding and enhance the survival of Crizotinib-treated Ba/F3 cells transfected with these EML4-ALK mutants^18^,^44^. Nonetheless, to our knowledge, direct evidence suggesting that ALK mutations can effectively decrease the binding between ALK and ALK inhibitors is lacking, and our results from studying Crizotinib-resistant Karpas 299 and SupM2 clones have provided the first direct evidence. Consistent with our concept that Crizotinib—ALK binding is a key determining factor of Crizotinib sensitivity, we found *ALK* gene mutations in both our generated Crizotinib-resistant cell clones derived from Karpas 299 and SupM2. Using CETSA, we had confirmed that both NPM-ALK mutants do not bind to Crizotinib at 52 °C.

With respect to the third factor that might regulate the interaction between Crizotinib and ALK, we hypothesize that the interaction between ALK and its binding partners may play a key role in influencing Crizotinib—ALK binding, and thus, Crizotinib-resistance. This hypothesis is based on a number of observations. First, 2 of the 4 Crizotinib-resistant cell lines included in this study, namely IMR32 and GOTO, are known to carry wild-type *ALK*. Thus, in addition to gene mutations of *ALK*, there are likely alternative mechanisms to promote Crizotinib resistance. Second, it has been published that cell lines carrying the same mutated ALK (e.g. F1174L in Kelly and LAN-1, both of which are neuroblastoma cell lines) displayed drastically different IC_50_ to Crizotinib^47^. Third, the interacting proteins of oncogenic tyrosine kinases have been shown to modulate resistance to tyrosine kinase inhibitors, although the exact mechanisms are unknown^48^,^49^,^50^,^51^,^52^. With this hypothesis, we made the observation that β-catenin, previously shown to be a binding partner of NPM-ALK^31^, is differentially expressed between Crizotinib-sensitive and –resistant cell lines. Importantly, siRNA knockdown of β-catenin significantly enhanced Crizotinib—ALK binding and the sensitivity to Crizotinib in Crizotinib-resistant cells. Using computation, we have collected evidence suggesting that the binding of β-catenin likely hinders the binding of Crizotinib to ALK, and this correlates well with our model. Another important consideration is that the resistance to Crizotinib in cells treated with β-catenin siRNA remained to be relatively high (i.e. IC_50_ ~300 nM). Thus, it is possible that other ALK-interacting proteins (yet to be identified) may continue to hinder the binding of Crizotinib to ALK, even in the absence of β-catenin. If these additional ALK-interacting proteins can be identified, simultaneous inhibition of these proteins along with β-catenin may further sensitize these cells to tyrosine kinase inhibitors. This new knowledge may underlie a novel approach in overcoming tyrosine kinase drug resistance. This approach may be particularly useful, considering the observation that siRNA knockdown of β-catenin was found to be effective even in cells with ALK mutations.

As mentioned above, the finding that exogenous NPM-ALK (via gene transfection) expressed in Crizotinib-resistant cell lines (IMR32 and SK-N-SH, Fig. 2) can effectively bind to Crizotinib has provided evidence that the structure of ALK is an important determinant of the Crizotinib—ALK binding. This finding also has raised another important consideration. Specifically, the observation that NPM-ALK expressed in these Crizotinib-resistant cells can effectively bind to Crizotinib, in spite of the high abundance of β-catenin in these cells, is intriguing. To explain this, we hypothesized that different ALK forms (thus different structures) showed different affinities for β-catenin. Results from Supplemental Figure 10 support this view. Specifically, immunoprecipitation of β-catenin pulled down a relatively large amount of ALK^F1174L^ or wild-type full-length ALK, but only a small portion of NPM-ALK transfected into these two Crizotinib-resistant cell lines (SK-N-SH and IMR32, respectively).

CETSA is a recently described method that has been shown to be useful in evaluating the clinical utility of new drugs^53^,^54^,^55^,^56^,^57^,^58^. To date, <15 studies using CETA have been published in the literature. Results from this study have strongly suggested that CETSA assay is a useful tool to predict Crizotinib sensitivity in ALK^+^ cancers. Compared to many other assays used to assess drug resistance, such as those measuring ATP binding by recombinant oncogenic proteins, the use of CETSA is advantageous in that the drug-target interactions are evaluated in a relevant cellular context. Whether CETSA can be used in the clinical setting to predict drug sensitivity probably needs large-scale studies employing clinical samples.

In conclusion, our study has provided novel insights into the mechanism underling the resistance to Crizotinib in ALK^+^ cancers. Our studies have provided direct evidence that Crizotinib—ALK interaction is the key determinant and predictor of Crizotinib sensitivity in these cancer cells. Furthermore, our finding that β-catenin as an ALK-binding protein can substantially contribute to Crizotinib resistance has opened a new avenue in overcoming the clinical resistance to tyrosine kinase inhibitors. Lastly, our data has suggested that further investigation of CETSA used in the clinical setting is warranted.

## Methods and Materials

### Cell lines

The characteristics of the ALK^+^ALCL cell lines (Karpas 299 and SupM2) have been previously described^59^. The ALK^+^ neuroblastoma cell lines (NB1, IMR32, GOTO and SK-N-SH) used in this study were kind gifts from Dr. Roseline Godbout (Department of Oncology, University of Alberta). The non-small cell lung cancer cell line, H2228, was a kind gift from Dr. Ming Tsao (Ontario Cancer Institute). The MCL cell line (SP53) has been previously described^60^. All cell lines were maintained in RPMI 1640 supplemented with 10% fetal bovine serum (FBS) (Life Technologies, Grand Island, NY, USA).

### Cellular thermal shift assay (CETSA)

The ability of compounds to interact with, and thereby stabilize the target in intact cells, was analysed essentially as described by Molina *et al*.^21^. Detailed protocol is provided in the Supplementary materials and methods.

### Reagents, Plasmids and siRNA transfection

Crizotinib (PF-2341066), was purchased from Sigma-Aldrich (Oakville, Ontario, Canada). The HSP90 inhibitor (NVP-AUY922) and Ceritinib (LDK378) were purchased from Selleck Chemicals. Doxorubicin was purchased from LC Laboratories (Woburn, MA, USA). Each compound was dissolved in DMSO for cell culture experiments. The pcDNA3-flag-ALK wild-type and ALK^F1174L^ were kindly provided by Dr. Junko Takita (The University of Tokyo, Tokyo, Japan)^61^. The EML4-ALK expression vector was a kind gift from Dr. James Dalton (University of Tennessee Health Science Center)^62^. The NPM-ALK expression vector was a kind gift from Dr. S. Morris (St. Jude Children’s Research Hospital)^63^. For the siRNA knockdown experiments, ALK and β-catenin specific ON-Target Plus SMARTpool small interfering RNA (siRNA) and scramble control were purchased from Thermo Scientific (Chicago, USA).

### Broad-Novartis Cancer Cell Line Encyclopedia

Gene expression data for CTNNB1 (β-catenin) was extracted from CCLE_Expression_Entrez_2012-10-18.res. We also extracted Crizotinib responsiveness for nine ALK-expressing cell lines^30^. Through the CCLE Terms of Access, we declare that, “those who carried out the original analysis and collection of the data bear no responsibility for the further analysis or interpretation of it.”

### Statistical analysis

All the statistical analyses were performed using the GraphPad Prism 5.1 program. Student *t* test was used to calculate *p* values. Results are presented as mean ± standard deviation. The Fisher’s exact test was used to correlate Crizotinib sensitivity with Crizotinib—ALK binding among the 7 ALK^+^ cancer cell lines. The nonparametric Spearman’s rank correlation coefficient was applied to evaluate the correlation between Crizotinib IC_50_ values and β-catenin mRNA levels.

## Additional Information

**How to cite this article**: Alshareef, A. *et al*. The use of cellular thermal shift assay (CETSA) to study Crizotinib resistance in ALK-expressing human cancers. *Sci. Rep.* **6**, 33710; doi: 10.1038/srep33710 (2016).

## Supplementary Material

Supplementary Information

## Figures and Tables

**Table 1:** Summary of the IC_50_ data (derived from the literature as well as our own studies) and the CETSA results.

Cancer type	Cell line	ALK form	IC_50_ (nM) (Standard Deviation)	Reported IC_50_ in nM (reference)	Crizotinib sensitivity	CETSA at 52 °C
ALCL	SupM2	*NPM-ALK*	40 (±9)	56^26^	Sensitive	Positive binding
	Karpas 299	*NPM-ALK*	45 (±7)	32^27^	Sensitive	Positive binding
Neuroblastoma	NB1	Full-length *ALK*^Amplified^	10 (±4)	10^28^	Sensitive	Positive binding
	IMR32	Full-length *ALK*^wild-type^	970 (±134)	740^29^	Resistant	Negative binding
	GOTO	Full-length *ALK*^wild-type^	1817 (±200)	>5000^30^	Resistant	Negative binding
	SK-N-SH	Full-length *ALK*^F1174L^	631 (±83)	1900^30^	Resistant	Negative binding
NSCLC	H2228	*EML4-ALK variant 3*	984 (±96)	834^29^	Resistant	Negative binding
