# Supplementary material for: The use of cellular thermal shift assay (CETSA) to study Crizotinib resistance in ALK-expressing human cancers
Source: Sci Rep. 2016 Sep 19;6:33710. doi: 10.1038/srep33710 (PMC5027386; doi:10.1038/srep33710)

Supplementary Data

The use of cellular thermal shift assay (CETSA) to study Crizotinib resistance in ALK-expressing human cancers

Abdulraheem Alshareef[1,2], Hai-Feng Zhang[1,3], Yung-Hsing Huang[1], Chengsheng Wu[1], JingDong Zhang[4], Peng Wang[5], Ahmed El-Sehemy[6], Mohamed Fares[7], Raymond Lai[1,8,9,*]

[1]Department of Laboratory Medicine and Pathology, University of Alberta, Edmonton, Alberta, Canada;
[2]Department of Applied Medical Sciences, Taibah University, Almedinah, P.O. Box 41477, Saudi Arabia;
[3]Department of Pathology and Laboratory Medicine, University of British Columbia, Vancouver, BC V5Z 1L3, Canada;
[4]Department of Medical Oncology, The First hospital of China Medical University, Shen Yang 110001, P.R. China;
[5]Department of Internal Medicine, University of Alberta, Edmonton, Alberta, Canada;
[6]Department of Laboratory Medicine and Pathobiology, University of Toronto, Toronto, Canada;
[7]National Research Center, Cairo, Egypt;
[8]Department of Oncology, University of Alberta, Edmonton, Alberta, Canada;
[9]DynaLIFEDx Medical Laboratories, Edmonton, Canada

***To whom correspondence should be addressed:**
Raymond Lai, MD, PhD, Department of Laboratory Medicine and Pathology, Cross Cancer Institute and University of Alberta, 11560 University Avenue, Room 2338, Edmonton, Alberta T6G 1Z2, Canada; E-mail: rlai@ualberta.ca.

Supplementary Figure 1

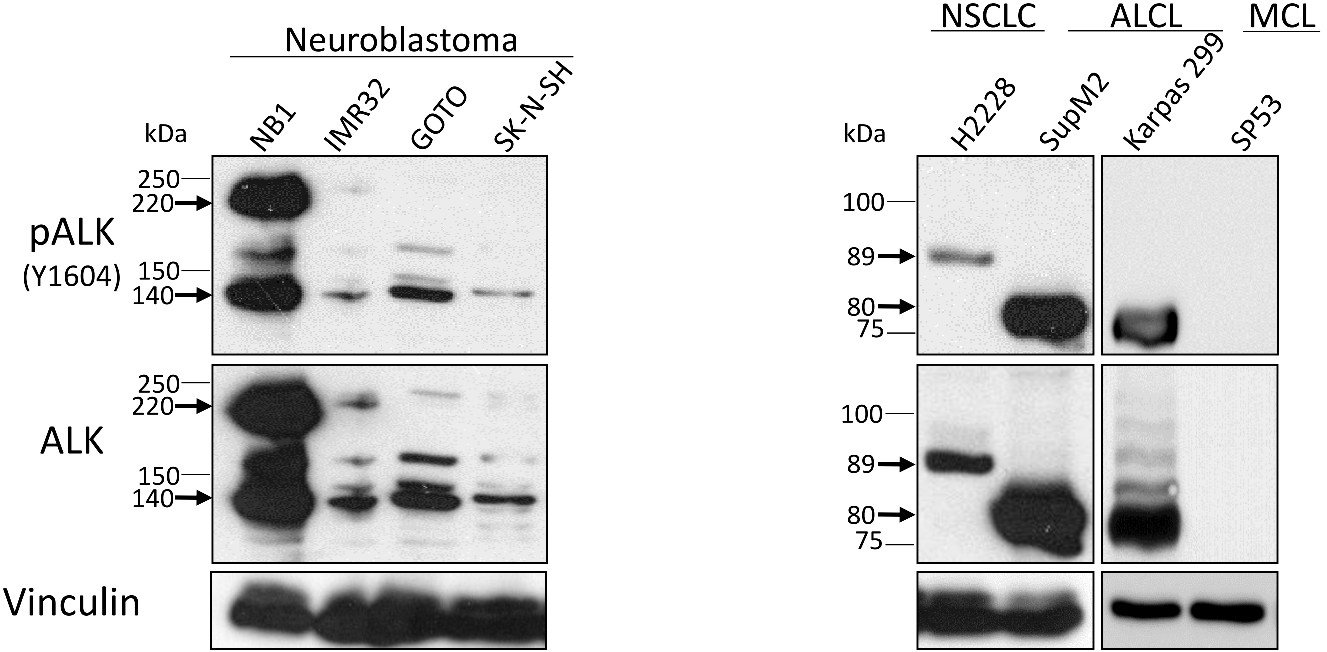

Supplementary Figure 2

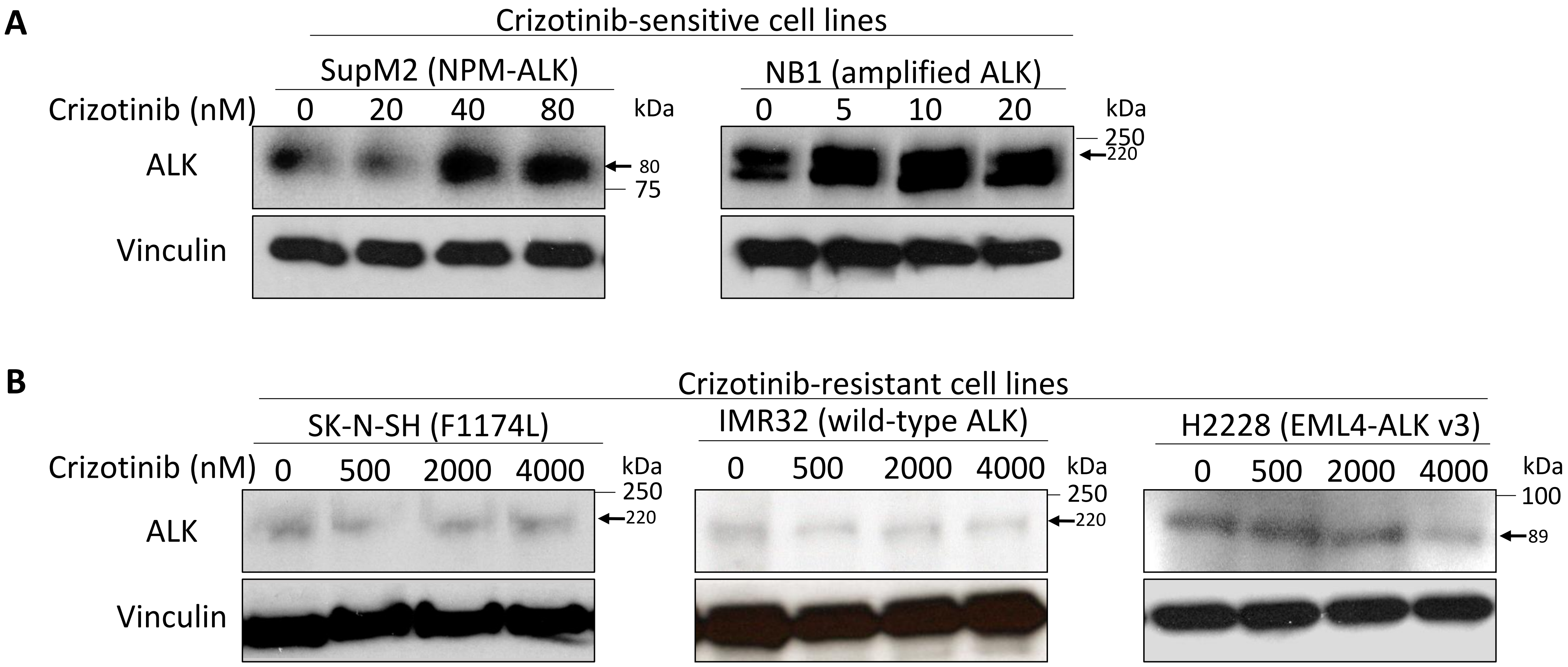

Supplementary Figure 3

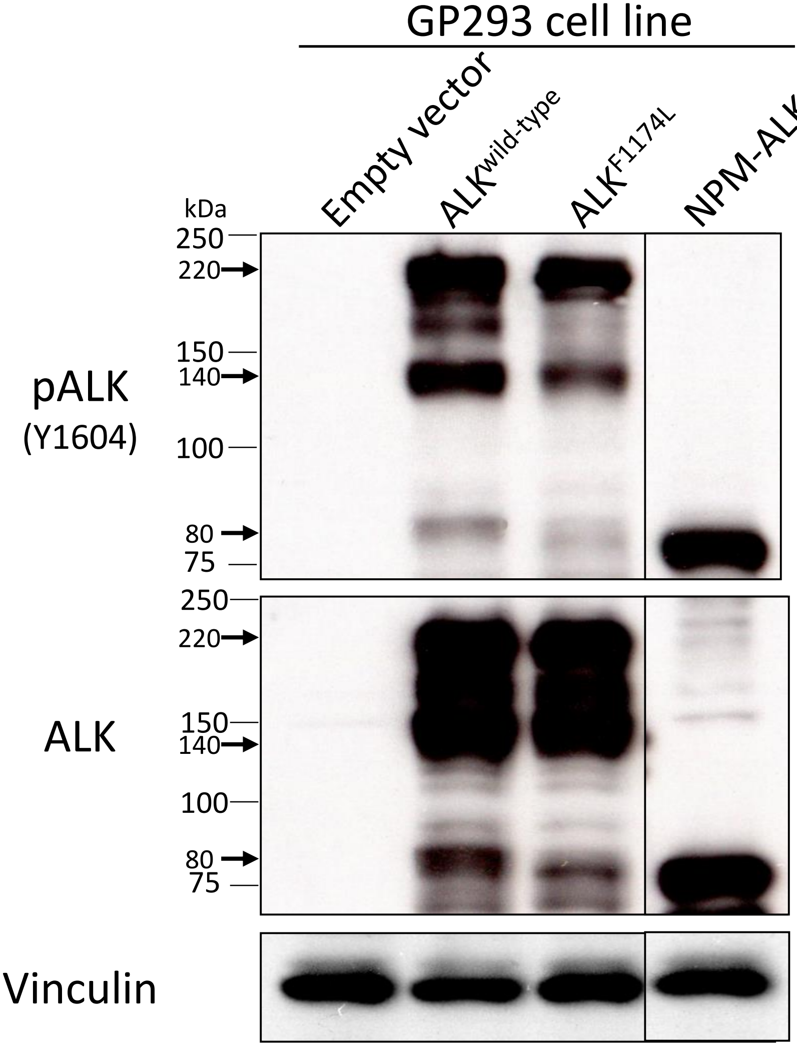

Supplementary Figure 4

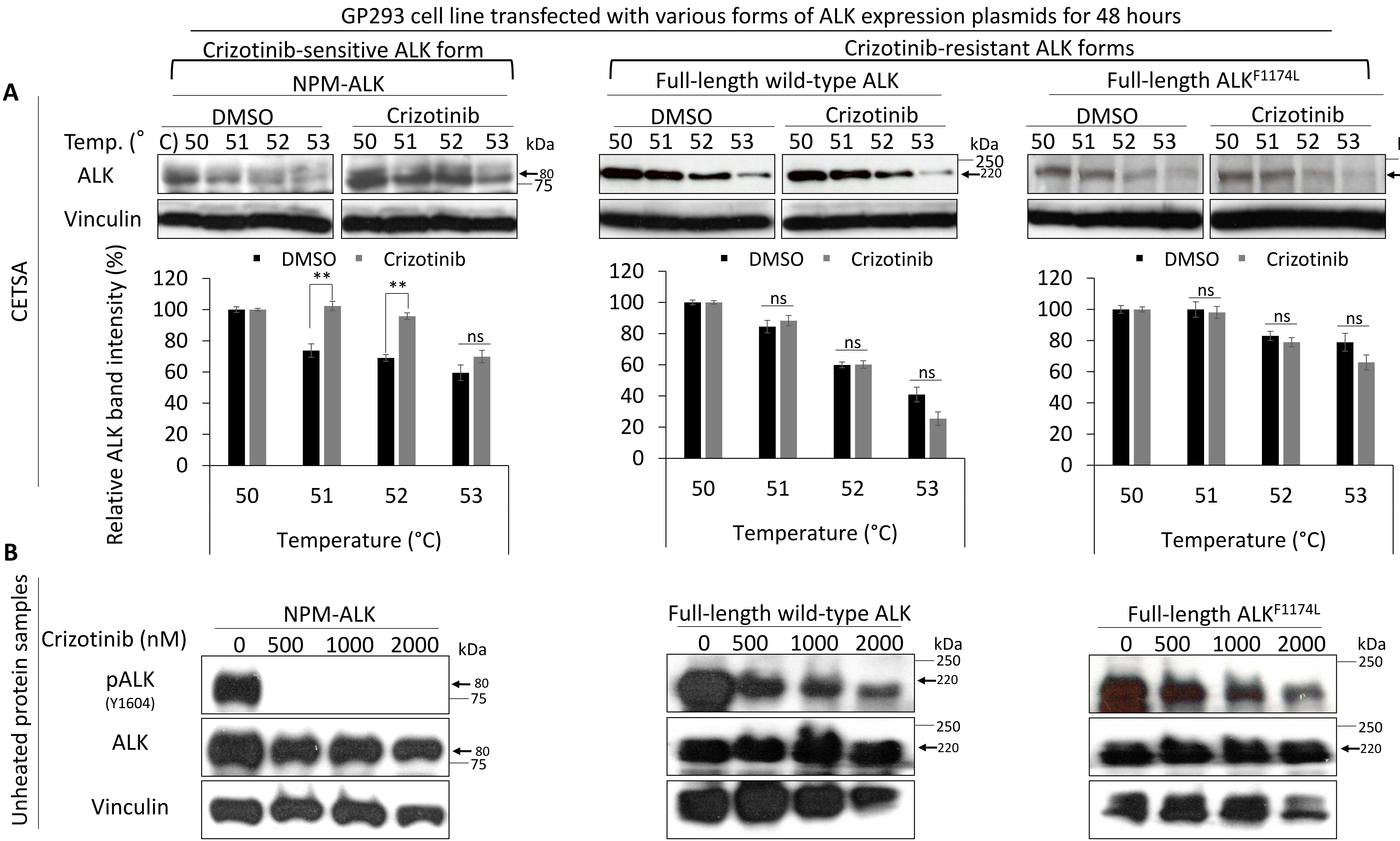

Supplementary Figure 5

A

Cell line	IC$_{50}$ in nM (Standard deviation)	
	Crizotinib	Ceritinib
Parental Karpas 299	45 (± 7)	29.3 (± 2)
Crizotinib-resistant Karpas 299 (G329A)	970 (± 23)	31.2 (± 3)
Fold	21.5	1.06

B

Cell line	IC$_{50}$ in nM (Standard deviation)	
	Crizotinib	Ceritinib
Parental SupM2	40 (± 9)	22.5 (± 2)
Crizotinib-resistant SupM2 (G262R)	1180 (± 27)	104.3 (± 12)
Fold	29.5	4.6

C

Karpas 299	Doxorubicin IC$_{50}$ in nM (Standard deviation)
Parental	63 (± 3)
Crizotinib -resistant	70 (± 4)
Fold	1.11

D

SupM2	Doxorubicin IC$_{50}$ in nM (Standard deviation)
Parental	72 (± 2)
Crizotinib -resistant	79 (± 5)
Fold	1.09

E

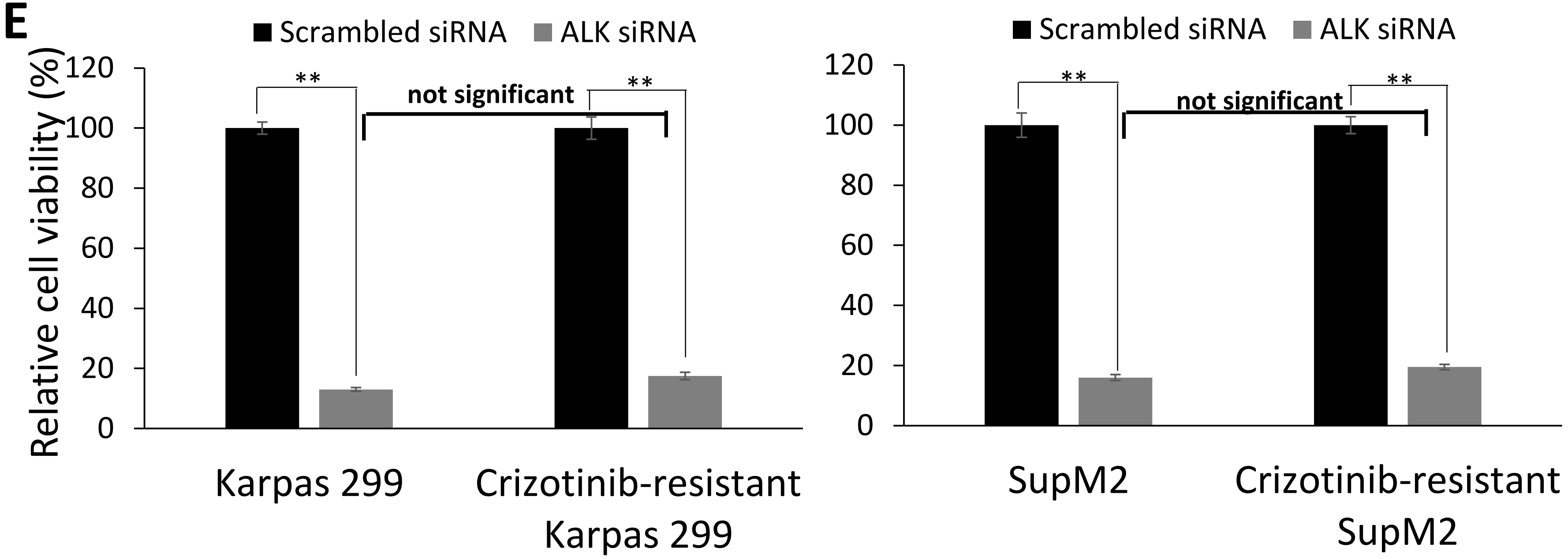

F

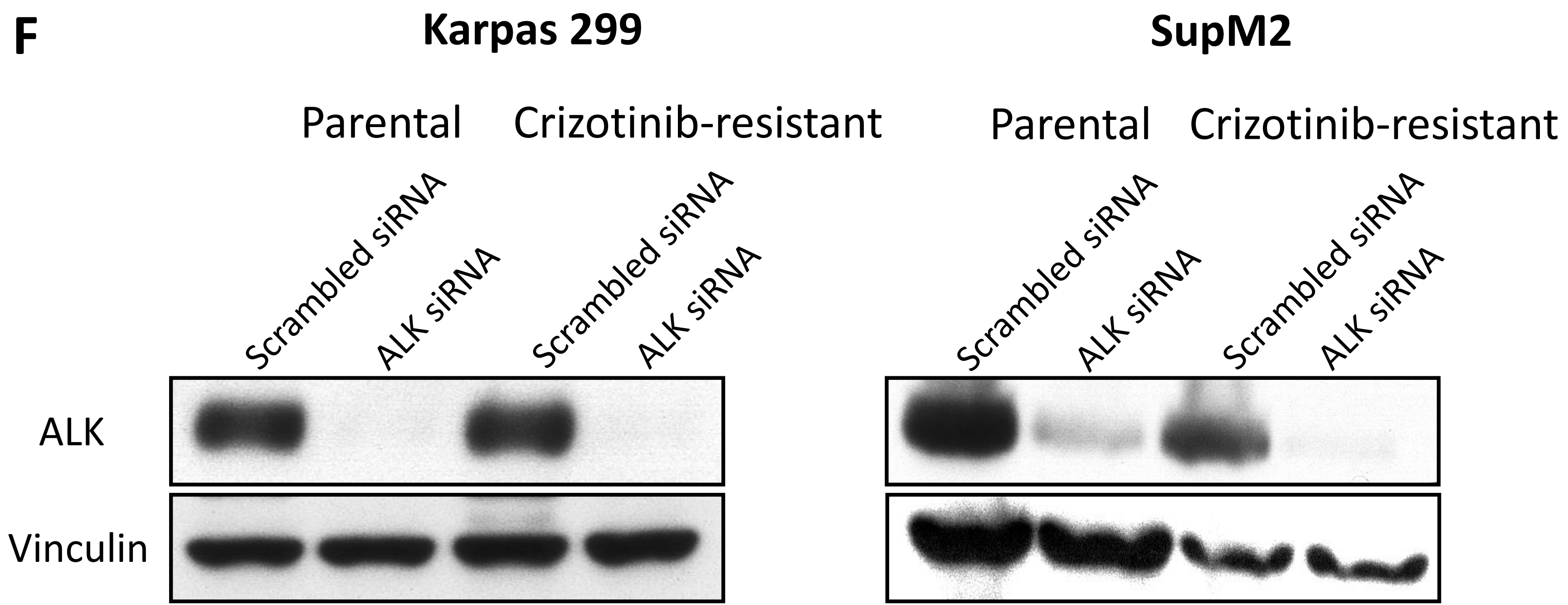

Supplementary Figure 6

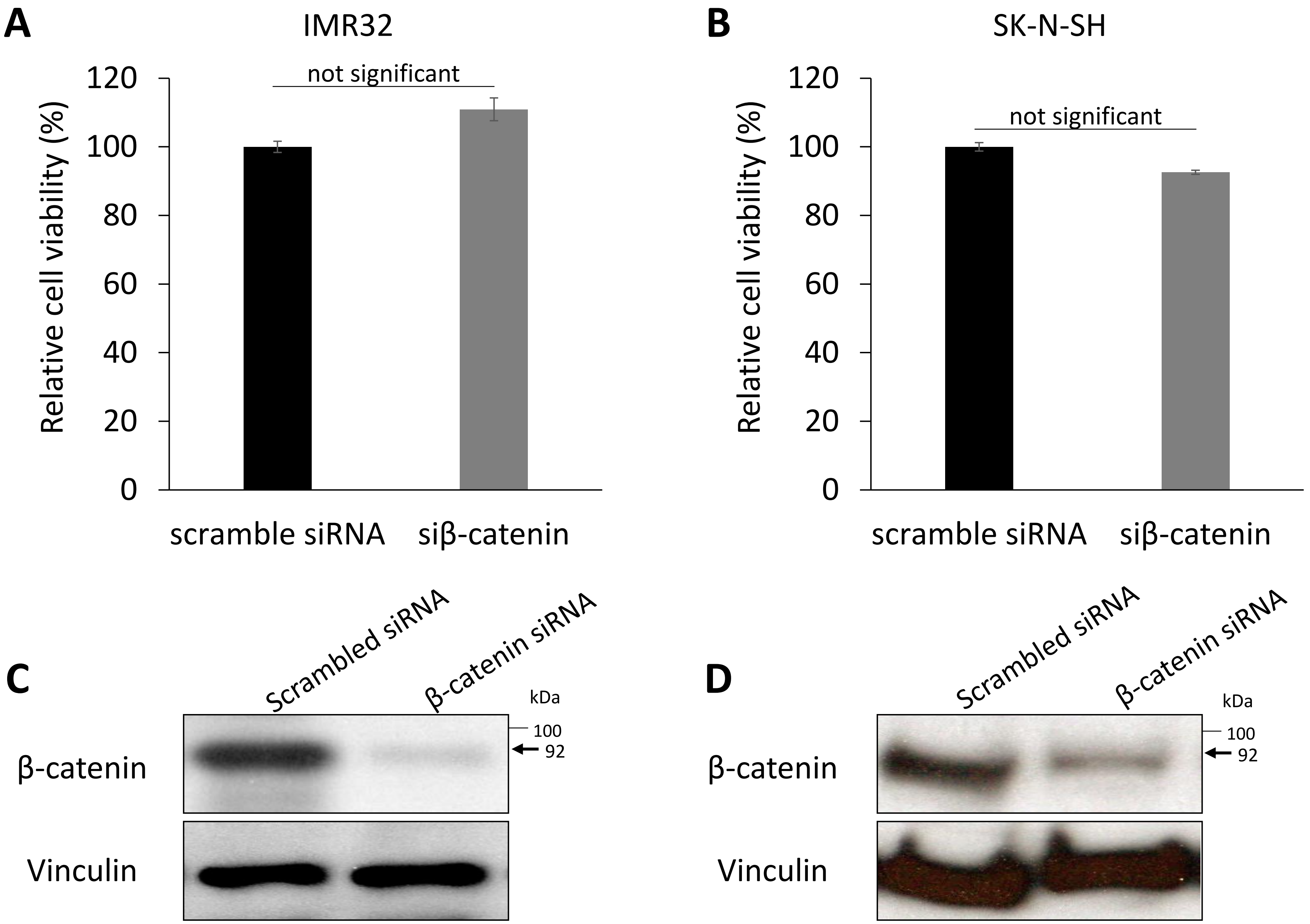

Supplementary Figure 7

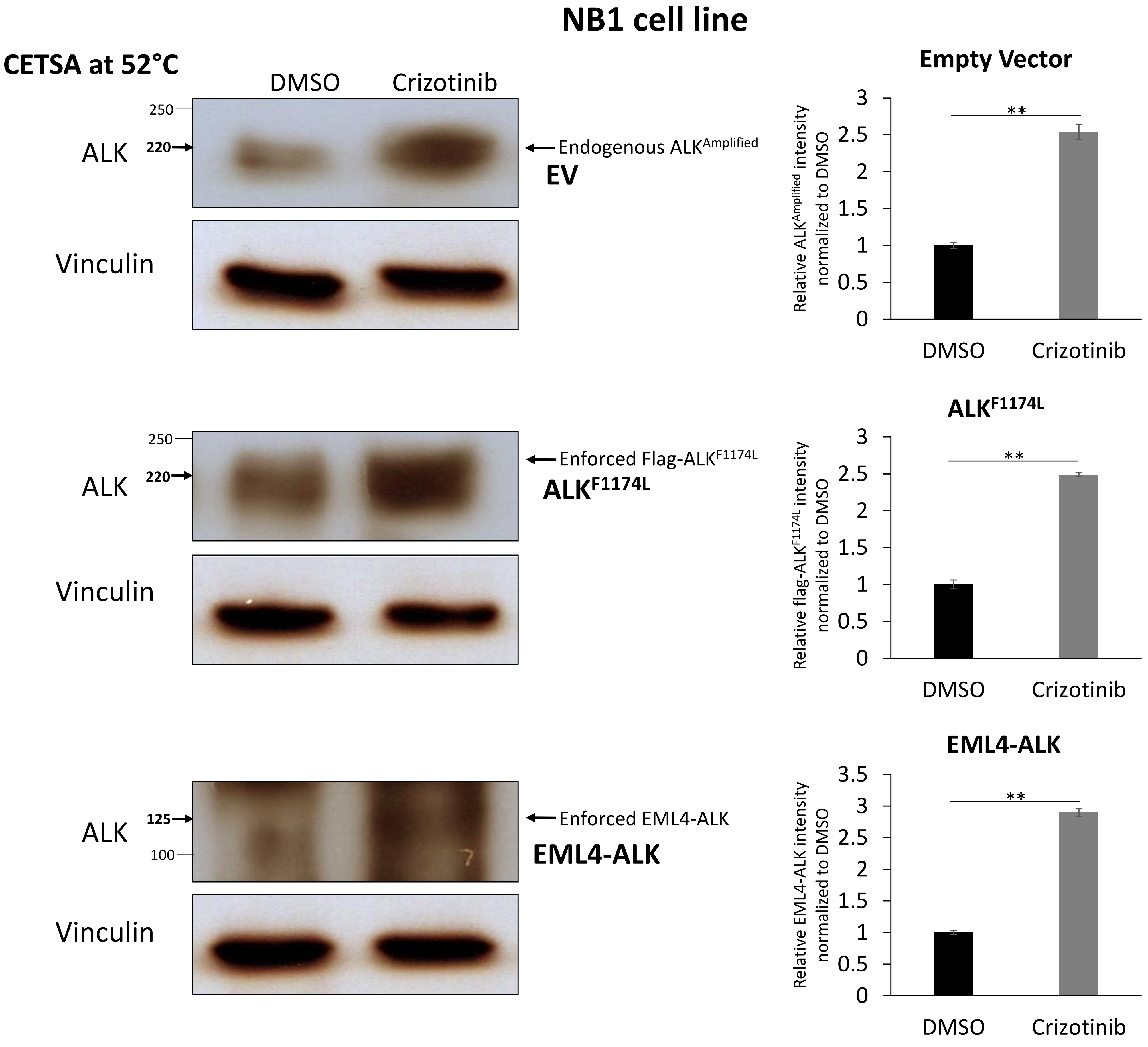

Supplementary Figure 8

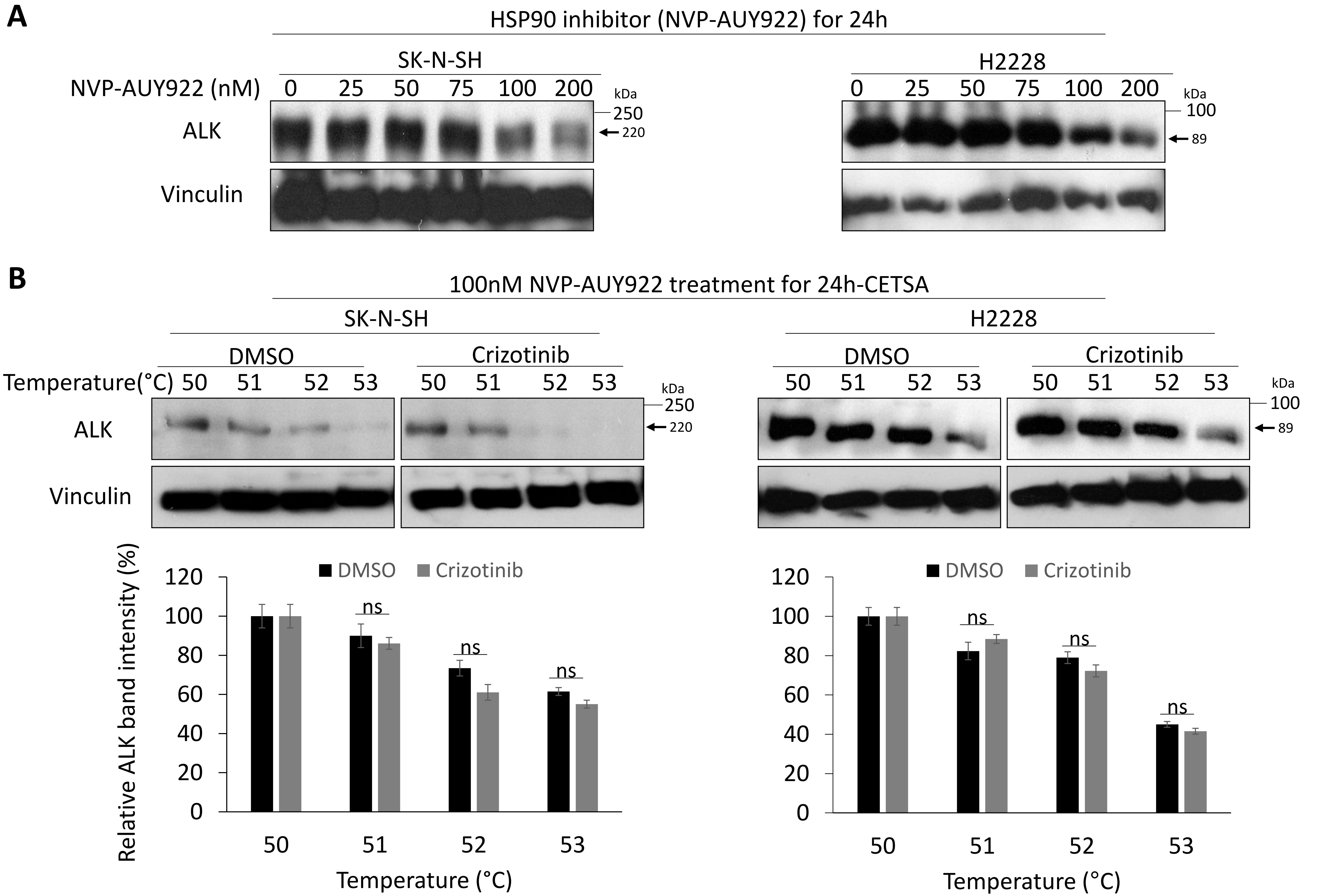

Supplementary Figure 9

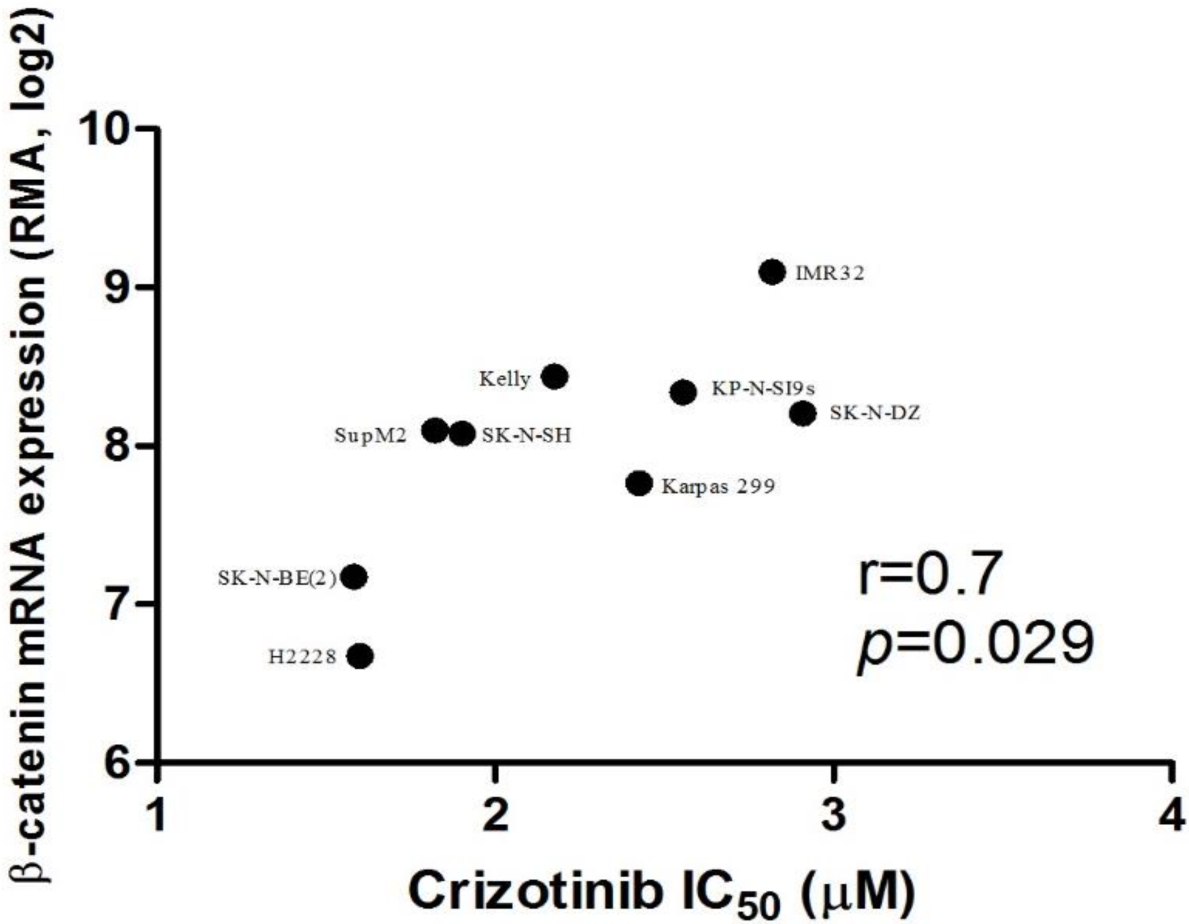

Supplementary Figure 10

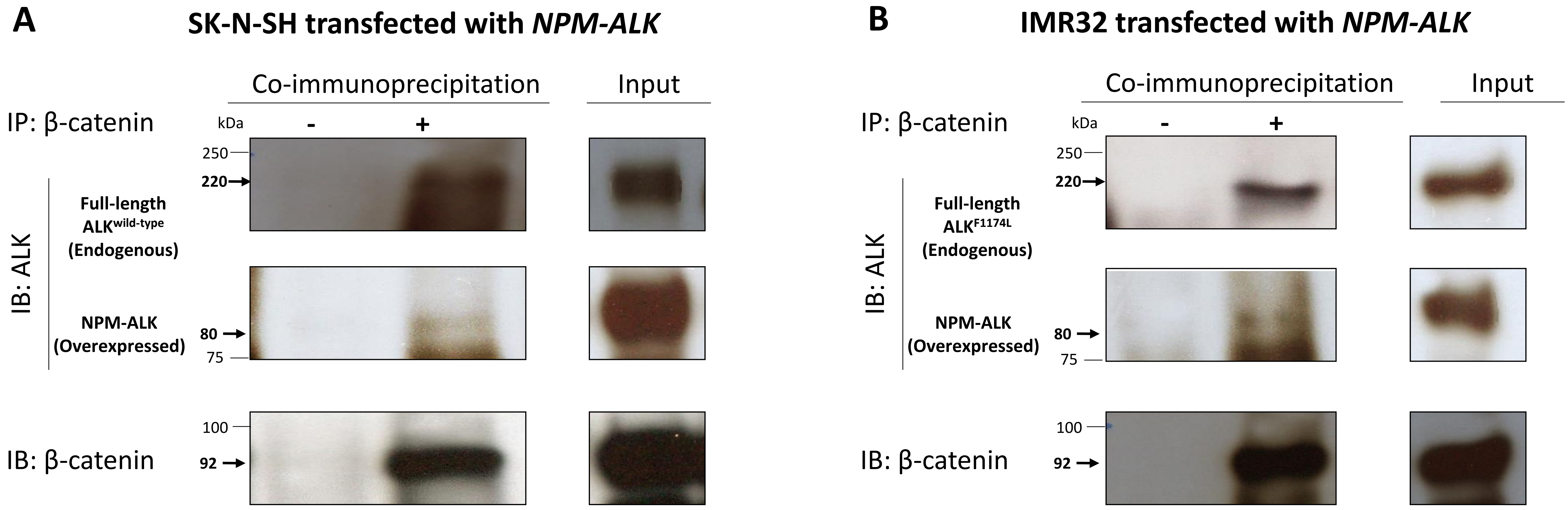

Supplementary Figure Legends

Supplementary Figure 1. Expression levels of pALK and ALK in 7 ALK-expressing cancer cell lines. The four ALK-positive neuroblastoma cell lines (NB1, IMR32, GOTO and SK-N-SH) express full-length ALK (220 kDa) and/or several bands at lower molecular weight (e.g. 140 kDa). The non-small cell lung cancer (NSCLC) cell line, H2228, expresses EML4-ALK translocation variant 3 (89 kDa). The two ALK^{+}ALCL cell lines (SupM2 and Karpas 299) express NPM-ALK (80 kDa). SP53, a mantle cell lymphoma (MCL) cell line, was used as a negative control and showed no ALK expression.

Supplementary Figure 2. Crizotinib binds to ALK in a dose-dependent manner in Crizotinib-sensitive cell lines while no binding is observed in Crizotinib-resistant cell lines. A. Two Crizotinib-sensitive cell lines (SupM2 and NB1) demonstrated a dose-dependent increase of stabilized ALK using CETSA assay at 52°C upon Crizotinib treatment. **B**. Three Crizotinib-resistant cell lines (SK-N-SH, IMR32 and H2228) showed no ALK stabilization when treated with Crizotinib at high dosages.

Supplementary Figure 3. Enforced expression of various forms of ALK-expression plasmids (full-length wild-type ALK, full-length ALK ^{F1174L} and wild-type NPM-ALK) in GP293 cells was determined by Western blots.

Supplementary Figure 4. The ALK structure is the main determinant of Crizotinib responsiveness and is correlated with inhibition of ALK phosphorylation upon enforced expression in the GP293 cell model. A. The CETSA assay results showed Crizotinib stabilized

NPM-ALK at 51°C and 52°C while unable to stabilize full-length wild-type ALK or full-length ALK^{F1174L} at the same temperatures. **B.** Crizotinib treatment completely abrogated ALK phosphorylation in NPM-ALK but not the other two forms of ALK, i.e. full-length wild-type ALK and full-length F1174L ALK. Data are presented as mean ± SD. **P<0.01, Student's *t* test.

Supplementary Figure 5. Crizotinib-resistant cell clones demonstrate similar pattern of growth inhibition as the parental cells. **A** and **B** show the no or a marginal fold change of Ceritinib IC_{50} for both the parental cells and Crizotinib-resistant cell clones (Karpas 299 and SupM2). **C** and **D** show the no fold change of doxorubicin IC_{50} for both the parental and Crizotinib-resistant cell clones. **E** shows a similar and dramatic reduction in cell viability in both the parental cells and Crizotinib-resistant cell clones upon siRNA knockdown of ALK. **F** shows representative Western blots after ALK siRNA knockdown in the parental cells and Crizotinib-resistant cell clones for both Karpas 299 and SupM2. Data are presented as mean ± SD. *P<0.05, **P<0.01, Student's *t* test.

Supplementary Figure 6. β-catenin knockdown does not affect the cell viability of IMR32 and SK-N-SH. **A** and **B** show the minimal effect of β-catenin siRNA knockdown on the cell viability of the two Crizotinib-resistant cell lines. **C** and **D** show representative Western blots after β-catenin knockdown in IMR32 and SK-N-SH cells.

Supplementary Figure 7. EML4-ALK and ALK^{F1174L} stabilized by Crizotinib in NB1 cells. Enforced expression of *EML4-ALK* or ALK^{F1174L} into NB1, a Crizotinib-sensitive cell line, were treated with 50 nM Crizotinib for 6 hours and showed stabilization of EML4-ALK and ALK^{F1174L}.

CETSA assay was performed at 52°C. Representative Western blots are shown on the left side and the densitometry quantification data from 3 independent experiments are shown on the right side. Data are presented as mean ± SD. *P<0.05, **P<0.01, Student's *t* test.

Supplementary Figure 8. HSP90 inhibition does not facilitate the Crizotinib—ALK binding in Crizotinib-resistant cell lines. A. NVP-AUY922 reduced the ALK expression level at 100nM in two Crizotinib-resistant cell lines. **B.** NVP-AUY922 treatment did not restore Crizotinib—ALK binding in the two Crizotinib-resistant cell lines.

Supplementary Figure 9. Positive correlation between β-catenin expression and the IC_{50} to Crizotinib treatment. Analysis of Crizotinib responsiveness amongst various ALK-expressing cell lines showing that Crizotinib IC_{50} significantly correlates with the β-catenin expression level (Spearman r =0.7, p=0.029).

Supplementary Figure 10. β-catenin interacts weakly with NPM-ALK transfected into Crizotinib-resistant cell lines. A. β-catenin pull-down experiment showed substantial ALK^{F1174L}-β-catenin binding but only a small portion of NPM-ALK-β-catenin binding. Right panel, the input for the co-immunoprecipitation. **B.** β-catenin pull-down experiment showed substantial $ALK^{wild\text{-}type}$-β-catenin binding but only a small portion of NPM-ALK-β-catenin binding. Right panel, the input for the co-immunoprecipitation.

Supplementary materials and methods

Cellular thermal shift assay (CETSA)

The ability of compounds to interact with, and thereby stabilize the target in intact cells, was analyzed essentially as described by Molina et al [1]. Briefly, cells cultured in 100 x 20 mm tissue culture dishes at 90% confluence were treated with media containing DMSO or Crizotinib (doses used as described in the text) for 6 hours. After treatment, cells were detached with trypsin, collected by centrifugation and subsequently resuspended in PBS. The cell suspension was aliquoted into four PCR tubes and heated for 3 minutes to 50, 51, 52 or 53°C. Subsequently, cells were lysed using liquid nitrogen and two repeated cycles of freeze-thaw. Precipitated proteins were separated from the soluble fraction by centrifugation at 17,000g for 20 minutes. Soluble proteins, collected in the supernatant, were kept at −80°C until Western blot analysis. Equal amount of proteins were loaded onto 6% SDS–PAGE gels, transferred to nitrocellulose membranes and analyzed using the ALK-antibody from Cell Signaling at a concentration of 1:1000. Protein expression levels on Western blots were quantified by densitometry analyses using the ImageJ software.

Cell viability analysis

Cell viability was performed as previously described [2,3]. The cell viability was then measured using the MTS assay (Promega, Madison, WI) following the manufacturer's protocol.

Immunoprecipitation and Western blot analysis

Western blotting, co-immunoprecipitation analyses and antibodies used were performed as described previously [4,5].

Molecular modeling and docking studies

PDB crystal structure files of ALK, Crizotinib [PDB ID: 2XP2] [6], β-catenin [PDB ID: 1LUJ] [7] and HSP90 [PDB ID: 1US7] [8] were downloaded from the public Research Collaboratory for Structural Bioinformatics Protein Data Bank (RCSB PDB) online database (www.rcsb.org/pdb) [9]. Protein structure files were handled using Pymol [10] and ICM-browser Molsoft [11,12] molecular editing programs in order to visualize the structures and prepare them for docking according to standard procedures; for every file the ligand was removed freeing the corresponding proteins of interest, then missing hydrogen atoms were added to the structure. For docking studies, we used Cluspro protein-protein online docking server provided by Boston University (http://cluspro.bu.edu/) according to the standard protocol and recommended specifications, and using cluster sizes to rank predicted binding models [13-16].

Reverse transcriptase-polymerase chain reaction (RT-PCR) and sequencing for secondary mutation

For RT-PCR, reverse transcription of mRNA was carried out using a protocol described previously [5]. The ALK primer sequences are as follows: forward) 5’-CTTTGGGGAGGTGTATGAAGG-3’ and reverse) 5’-GGCACTTTCTCTTCCTCTTCC-3’. The expected PCR product size is 858 bp. Sequencing was performed at The Applied Genomics Centre (TAGC), University of Alberta.

References:

1 Martinez Molina, D. *et al.* Monitoring drug target engagement in cells and tissues using the cellular thermal shift assay. *Science* **341**, 84-87 (2013).

2 Zhang, J. *et al.* Aberrant expression of the transcriptional factor Twist1 promotes invasiveness in ALK-positive anaplastic large cell lymphoma. *Cellular Signalling* **24**, 852-858 (2012).

3 Anand, M., Lai, R. & Gelebart, P. beta-catenin is constitutively active and increases STAT3 expression/activation in anaplastic lymphoma kinase-positive anaplastic large cell lymphoma. *Haematologica* **96**, 253-261 (2011).

4 Hegazy, S. A. *et al.* Disheveled proteins promote cell growth and tumorigenicity in ALK-positive anaplastic large cell lymphoma. *Cell Signal* **25**, 295-307 (2013).

5 Wu, C. *et al.* STAT1 is phosphorylated and downregulated by the oncogenic tyrosine kinase NPM-ALK in ALK-positive anaplastic large-cell lymphoma. *Blood* **126**, 336-345 (2015).

6 Cui, J. J. *et al.* Structure based drug design of crizotinib (PF-02341066), a potent and selective dual inhibitor of mesenchymal-epithelial transition factor (c-MET) kinase and anaplastic lymphoma kinase (ALK). *Journal of medicinal chemistry* **54**, 6342-6363 (2011).

7 Graham, T. A., Clements, W. K., Kimelman, D. & Xu, W. The crystal structure of the beta-catenin/ICAT complex reveals the inhibitory mechanism of ICAT. *Molecular cell* **10**, 563-571 (2002).

8 Roe, S. M. *et al.* The Mechanism of Hsp90 regulation by the protein kinase-specific cochaperone p50(cdc37). *Cell* **116**, 87-98 (2004).

9 Berman, H. M. *et al.* The Protein Data Bank. *Nucleic Acids Research* **28**, 235-242, www.rcsb.org/pdb (2000) (Date of access: 03/12/2015).

10 Schrodinger, LLC. *The PyMOL Molecular Graphics System, Version 1.3r1* (2010).

11 Abagyan, R. & Totrov, M. Biased probability Monte Carlo conformational searches and electrostatic calculations for peptides and proteins. *Journal of molecular biology* **235**, 983-1002 (1994).

12 Abagyan, R., Totrov, M. & Kuznetsov, D. ICM—A new method for protein modeling and design: Applications to docking and structure prediction from the distorted native conformation. *Journal of Computational Chemistry* **15**, 488-506 (1994).

13 Kozakov, D. *et al.* How good is automated protein docking? *Proteins* **81**, 2159-2166 (2013).

14 Kozakov, D., Brenke, R., Comeau, S. R. & Vajda, S. PIPER: an FFT-based protein docking program with pairwise potentials. *Proteins* **65**, 392-406 (2006).

15 Comeau, S. R., Gatchell, D. W., Vajda, S. & Camacho, C. J. ClusPro: an automated docking and discrimination method for the prediction of protein complexes. *Bioinformatics (Oxford, England)* **20**, 45-50 (2004).

16 Comeau, S. R., Gatchell, D. W., Vajda, S. & Camacho, C. J. ClusPro: a fully automated algorithm for protein-protein docking. *Nucleic Acids Res* **32**, W96-99 (http://cluspro.bu.edu/) (2004) (Date of access: 20/12/2015).

Supplementary Figure 11. Uncropped images of the blots presented in the main figure 1.

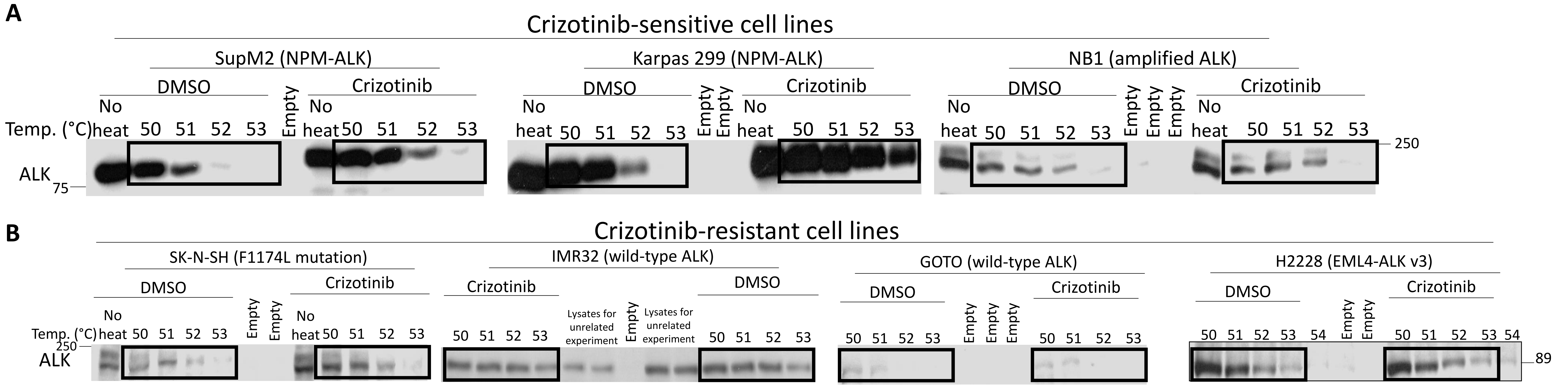

Supplementary Figure 12. Uncropped images of the blots presented in the main figure 3.

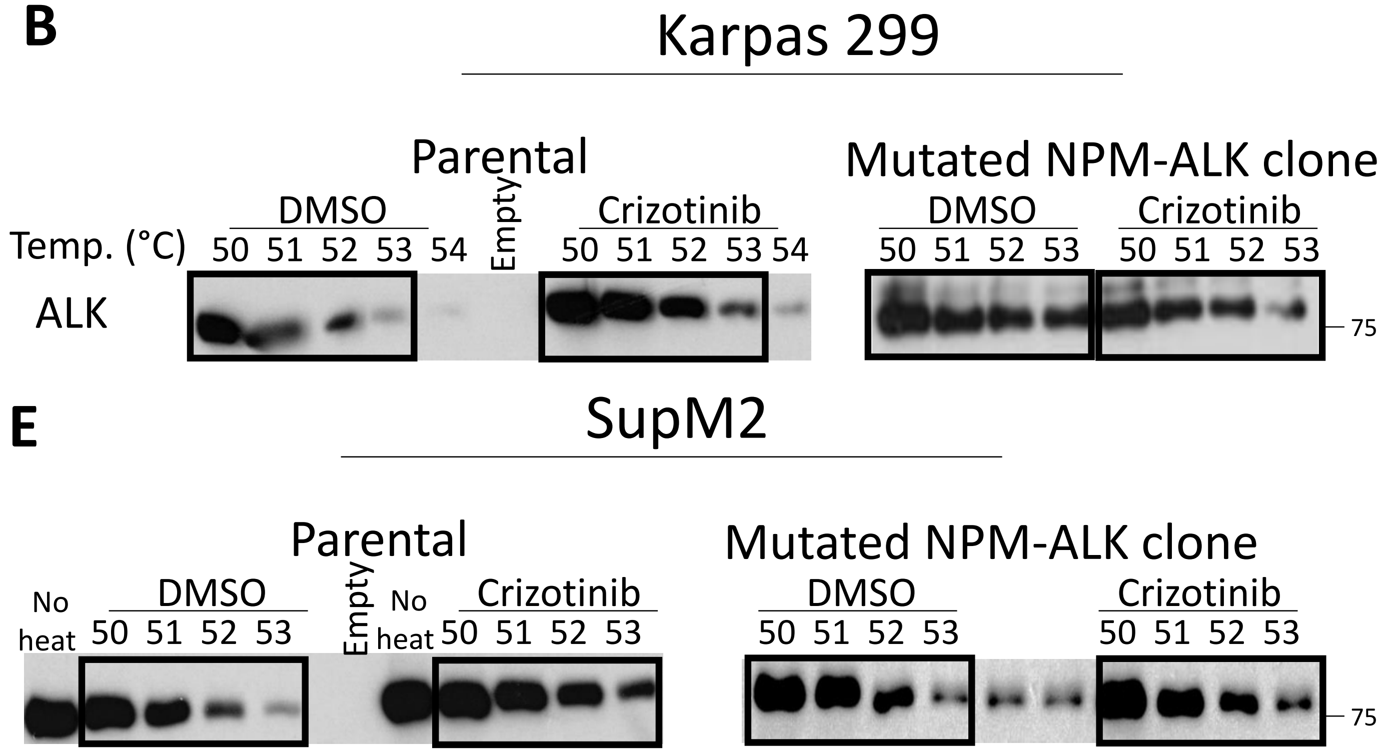

Supplement: Supplementary Information [file srep33710-s1.pdf]
